# Cinnarizine vs. topiramate for migraine prophylaxis in children: a randomized, double-blind, parallel-group clinical trial

**DOI:** 10.1186/s12883-026-04950-1

**Published:** 2026-05-09

**Authors:** Alireza Abdi, Ezatollah Abbasi, Aisan Abazarlou

**Affiliations:** Department of pediatric diseases, Faculty of Medicine, Urmia University of Medical Sciences, Urmia, Iran

**Keywords:** Cinnarizine, Efficacy, Migraine, Migraine Prophylaxis, Pediatric, Topiramate, Tolerability

## Abstract

**Objectives:**

Migraine is a common and disabling neurological disorder in children, often requiring preventive treatment. This study aimed to compare the efficacy and tolerability of cinnarizine and topiramate in the prophylaxis of migraine in children and adolescents.

**Materials and methods:**

In this randomized, double-blind, parallel-group clinical trial, 96 children aged 6–15 years with a diagnosis of migraine according to the International Classification of Headache Disorders (ICHD-3) [1], including both migraine with and without aura, without restriction to specific subtypes were enrolled. Participants were randomly assigned to receive either cinnarizine (25 mg once daily) or topiramate (25 mg once daily) for 12 weeks. Migraine attack frequency and severity were recorded using headache diaries at baseline, one month, and three months after treatment initiation. The primary outcome was the change in monthly migraine attack frequency. Secondary outcomes included changes in headache severity and the occurrence of adverse events. Between-group comparisons were performed using the Mann–Whitney U test, and within-group changes over time were analyzed using the Friedman test.

**Results:**

Both cinnarizine and topiramate significantly reduced monthly migraine attack frequency and headache severity over the 12-week treatment period (*P* < 0.001 for within-group comparisons). At three months, the median number of migraine attacks decreased to two attacks per month in both groups, with no statistically significant difference observed between the two treatments (*P* = 0.81). Headache severity scores also improved significantly in both groups, and no between-group differences were observed at follow-up (*P* = 0.74). Both medications were generally well tolerated, with no statistically significant differences in the incidence of adverse events between groups. Appetite reduction was reported in 4.2% of the cinnarizine group versus 14.6% of the topiramate group. While a numerically lower incidence of appetite reduction was observed in the cinnarizine group (4.2% vs. 14.6%), this difference was not statistically significant (*P* = 0.159, Fisher’s exact test; OR = 0.255, 95% CI: 0.05–1.27).

**Conclusion:**

Both cinnarizine and topiramate significantly reduced the frequency and severity of migraine attacks in children. No statistically significant difference in efficacy was detected between the two treatments. A numerically lower incidence of appetite reduction was observed with cinnarizine, although this difference was not statistically significant. Larger studies are needed to evaluate potential differences in tolerability.

**Trial registration:**

Iranian Registry of Clinical Trials (IRCT) IRCT20221108056444N1. Registered 12 February 2023 Retrospectively registered, https://www.en.irct.ir/trial/66774.

**Supplementary Information:**

The online version contains supplementary material available at 10.1186/s12883-026-04950-1.

## Introduction

Migraine is among the most common neurological disorders affecting children and adolescents, significantly affecting their quality of life, academic performance, and psychological wellbeing. It is characterized by recurrent episodes of moderate-to-severe headaches, often accompanied by nausea, vomiting, photophobia, and phonophobia, leading to substantial disability in the pediatric population [2]. Unlike adults, children may experience shorter headache durations and more diffuse symptoms, which complicates diagnosis and management [3, 4].

Epidemiological studies suggest that approximately 11% of children and adolescents suffer from migraine, with a notable increase in prevalence during adolescence, particularly among females [5]. Recent studies report migraine prevalence in approximately 7.7% to 9.1% of children, with higher rates in older age groups [6, 7]. In Iran, a study conducted among schoolchildren in Yazd found that 6.1% of the children experienced migraine [8]. These statistics underscore the significant public health impact of pediatric migraine and highlight the necessity of developing effective preventive strategies.

Current management approaches for pediatric migraine include both acute and preventive strategies. Acute treatment typically involves analgesics, such as acetaminophen, and nonsteroidal anti-inflammatory drugs (NSAIDs), such as ibuprofen. For more severe attacks, triptans such as rizatriptan and sumatriptan are used, with certain formulations approved for use in children over specific age thresholds [9, 10]. Preventive therapy is considered for children with frequent or debilitating migraine. Topiramate, an anticonvulsant, is effective in reducing the frequency and severity of migraine attacks in children. It operates through several mechanisms, including the inhibition of voltage-gated sodium channels, enhancement of GABAergic activity, and antagonism of glutamate receptors. While effective, it may be associated with side effects, such as cognitive impairment, weight loss, and paresthesia, as well as its well-known teratogenic potential, which is particularly relevant in adolescent females [11, 12]. Cinnarizine, an antihistamine and calcium channel blocker, has also shown promise for migraine prophylaxis. It is believed to modulate the cerebral blood flow and reduce the excitability of the vestibular system. Compared with topiramate, cinnarizine is generally better tolerated, with fewer cognitive side effects. However, its efficacy relative to topiramate in pediatric migraine prophylaxis is less well established and warrants further investigation [13, 14].

This study aimed to compare the efficacy and tolerability of cinnarizine and topiramate in the prophylaxis of migraine in children. By evaluating headache frequency, duration, severity, and adverse events, this study aimed to provide evidence-based recommendations for pediatric neurologists. Ultimately, the findings of this study could contribute to the development of evidence-based guidelines for pediatric migraine management in Iran and other similar regions, thereby improving treatment outcomes and quality of life for affected children.

## Materials and methods

### Study design and setting

This randomized, double-blind, parallel-group clinical trial was designed to compare the efficacy and tolerability of cinnarizine and topiramate for migraine prophylaxis in children. The study was conducted at the Pediatric Neurology Clinic of Urmia University of Medical Sciences, Urmia, Iran, during 2023. The trial protocol was developed in accordance with the Consolidated Standards of Reporting Trials (CONSORT) guidelines (Supplementary file 1).

### Participants

Children aged 6 to 15 years with a confirmed diagnosis of migraine were considered eligible for participation in this study. Migraine was diagnosed according to the International Classification of Headache Disorders, third edition (ICHD-3). Eligible participants were required to have a documented history of frequent or disabling migraine attacks, defined as at least one migraine episode per week during the six months preceding enrollment, and to be deemed candidates for preventive therapy by a pediatric neurologist. Written informed consent was obtained from the parents or legal guardians of all participants prior to inclusion in the study.

Children were excluded if they had evidence of secondary headache disorders, abnormal findings on neuroimaging, or had received any migraine prophylactic medications within three months prior to enrollment. Neuroimaging (CT or MRI) was not performed routinely for all patients. It was reserved only for those subjects presenting with ‘red flag’ signs that suggested a secondary headache disorder, in accordance with standard practice guidelines for pediatric migraine. Additional exclusion criteria included known hypersensitivity or contraindications to cinnarizine or topiramate, the presence of significant neurological, psychiatric, hepatic, or renal comorbidities, and inability of the child or caregiver to comply with study procedures or scheduled follow-up visits.

### Sample size calculation

The sample size was calculated based on detecting a moderate difference in the reduction of monthly migraine attack frequency between the two treatment groups. Assuming a two-sided significance level of 0.05, a statistical power of 80%, and an effect size (Cohen’s d) of 0.6, a minimum of 44 participants per group was required. To compensate for a potential 10% dropout rate, the final target sample size was increased to 48 participants per group, resulting in a total planned sample size of 96 children and adolescents.

### Randomization and allocation

Participants were randomly assigned in a 1:1 ratio to receive either cinnarizine or topiramate using block randomization with variable block sizes to ensure balanced group allocation. The randomization sequence was generated by an independent researcher not involved in patient recruitment or assessment. Allocation concealment was maintained using sealed, opaque, sequentially numbered envelopes. Medications were identical in appearance to ensure double-blinding of participants, parents, and investigators.

### Interventions

Participants in the cinnarizine group received cinnarizine 25 mg orally once daily at bedtime, while participants in the topiramate group received topiramate 25 mg orally once daily at bedtime. The treatment duration for both groups was 12 weeks.

The selected dosages were based on commonly used clinical doses in pediatric migraine prophylaxis and prior clinical trials. No dose escalation was performed during the study period. The fixed dosing regimen was chosen to simplify the protocol, although this approach is a recognized limitation as it may lead to suboptimal dosing for older or heavier children and adolescents. Concomitant use of acute analgesic medications for breakthrough migraine attacks was permitted and recorded.

### Outcome measures

The primary outcome of this study was the change in monthly migraine attack frequency from baseline to the end of the 12-week treatment period. Migraine attacks were defined as discrete headache episodes fulfilling ICHD-3 criteria for migraine (a single attack may last 4–72 h). Although monthly migraine days is a preferred standardized endpoint in many contemporary trials, attack frequency was used here as per protocol.

Secondary outcomes included changes in headache severity and headache frequency categories over time, as well as the incidence and type of treatment-related adverse events. Headache severity was classified into three categories: mild headaches, defined as those not interfering with daily activities; moderate headaches, defined as those partially interfering with daily activities; and severe headaches, defined as those causing significant functional impairment or requiring rest or medical intervention. This 3-point severity scale (mild/moderate/severe), while practical for diary use, is not formally validated and should be considered exploratory. Future studies should incorporate validated tools such as the Pediatric Migraine Disability Assessment (PedMIDAS) or visual analog scales (VAS). The use of this non-validated tool is a limitation that may reduce the precision of the severity outcome assessment. Adverse events were systematically assessed at each follow-up visit using a structured questionnaire that inquired about predefined common symptoms (including fatigue, sedation, drowsiness, appetite changes, paresthesia, and cognitive complaints) as well as open-ended reporting of any other symptoms.

### Data collection and follow-up

Baseline demographic and clinical data were collected using a structured questionnaire (Supplementary File 2) at the time of enrollment. Collected information included age, sex, clinical characteristics of migraine, and relevant medical history. Participants and their parents were instructed on how to complete a headache diary, which was used to prospectively document headache frequency, duration, severity, and associated symptoms throughout the study period.

Follow-up assessments were conducted at three predefined time points: at baseline prior to treatment initiation, one month after the initiation of therapy, and three months after the initiation of therapy. At each follow-up visit, headache diaries were reviewed, and data regarding headache frequency and severity were recorded. Additionally, participants were evaluated for the occurrence of adverse events related to the study medications. Compliance with treatment was assessed through caregiver reports and review of medication usage. All collected data were recorded by the research team in a standardized manner to ensure consistency and accuracy.

### Statistical analysis

Statistical analyses were performed using SPSS software (version 21). The normality of continuous variables was assessed using the Kolmogorov–Smirnov test. Normally distributed variables were expressed as mean ± standard deviation, while non-normally distributed variables were reported as median with interquartile ranges (IQR). Categorical variables were reported as frequencies and percentages.

Between-group comparisons of ordinal outcomes (headache severity and frequency categories) were performed using the Mann–Whitney U test. Within-group changes over time were analyzed using the Friedman test. Categorical variables were compared using Fisher’s exact test where appropriate.

Effect sizes were calculated to complement p-values, including rank-biserial correlation for Mann–Whitney U tests and odds ratios with 95% confidence intervals for categorical outcomes. All tests were two-sided. A *p*-value < 0.05 was considered statistically significant.

### Ethical considerations

The study protocol was approved by the Ethics Committee of Urmia University of Medical Sciences (IR.UMSU.REC.1401.277) and registered in the Iranian Clinical Trials Registry (IRCT20221108056444N1; registered on 2023-02-12- Retrospectively registered). Written informed consent was obtained from the parents or legal guardians of all participants prior to enrollment. Participant confidentiality was strictly maintained throughout the study, and patients were free to withdraw at any time without consequences.

## Results

### Participant flow and baseline characteristics

A total of 96 children and adolescents with a confirmed diagnosis of migraine were enrolled and randomized into two treatment groups, with 48 participants allocated to the cinnarizine group and 48 participants to the topiramate group. All randomized participants completed the 12-week follow-up period and were included in the final analysis.

The mean age of the study population was 10.0 ± 2.1 years (range: 6–15 years), with no significant difference between the cinnarizine and topiramate groups (9.9 ± 2.0 vs. 10.1 ± 2.2 years, respectively; *P* = 0.61, independent samples t-test). Overall, 59 participants (61.5%) were male and 37 (38.5%) were female, with comparable sex distribution between the two groups (*P* = 1.0, Fisher’s exact test). No statistically significant differences were observed between the groups regarding family history of migraine or duration of migraine symptoms at baseline (*P* > 0.05 for all comparisons) (Table 1). No participants had significant pre-existing comorbidities (as per exclusion criteria), and none were receiving concomitant prophylactic medications at baseline. Concomitant use of acute analgesics for breakthrough attacks was permitted and recorded, with comparable usage between groups.

**Table 1:** Baseline demographic and clinical characteristics of participants

Variable	Cinnarizine (*n* = 48)	Topiramate (*n* = 48)	Total (*n* = 96)	*P*-value
Age (years), mean ± SD	9.9 ± 2.0	10.1 ± 2.2	10.0 ± 2.1	0.61*
Body weight (kg), mean ± SD	32.4 ± 8.7	33.1 ± 9.2	32.8 ± 8.9	0.68*
Sex, n (%)				1.0**
Male	30 (62.5%)	29 (60.4%)	59 (61.5%)	
Female	18 (37.5%)	19 (39.6%)	37 (38.5%)	
Family history of migraine, n (%)				0.793**
Yes	41 (85.4%)	39 (81.2%)	80 (83.3%)	
No	7 (14.6%)	9 (18.8%)	16 (16.7%)	
Duration of migraine symptoms, n (%)				0.922***
< 6 months	8 (16.7%)	7 (14.6%)	15 (15.6%)	
6–12 months	21 (43.8%)	20 (41.7%)	41 (42.7%)	
> 12 months	19 (39.6%)	21 (43.8%)	40 (41.7%)	

*Independent samples t-test

**Fisher’s exact test

***Chi-square test

### Primary outcome: monthly migraine attack frequency

At baseline, the median monthly migraine attack frequency was comparable between the two groups (cinnarizine: median 7 attacks/month [IQR: 5–9]; topiramate: median 7 attacks/month [IQR: 5–8]; *P* = 0.72, Mann–Whitney U test).

Both treatment groups demonstrated a significant reduction in monthly migraine attack frequency over the 12-week treatment period. In the cinnarizine group, the median frequency decreased from 7 attacks/month at baseline to 4 attacks/month at one month and further to 2 attacks/month at three months (*P* < 0.001, Friedman test). Similarly, in the topiramate group, the median frequency decreased from 7 attacks/month at baseline to 4 attacks/month at one month and to 2 attacks/month at three months (*P* < 0.001, Friedman test).

Between-group comparisons showed no statistically significant difference in migraine attack frequency at one month (*P* = 0.68) or at three months (*P* = 0.81). The effect size for the between-group comparison at three months was small (rank-biserial correlation *r* = 0.06), indicating that no significant difference in efficacy was detected between the two treatments (Table 2).

**Table 2:** Monthly migraine attack frequency at baseline, one month, and three months

Time point	Cinnarizine (*n* = 48) Median (IQR)	Topiramate (*n* = 48) Median (IQR)	*P*-value^†^
Baseline, median (IQR)	7 (5–9)	7 (5–8)	0.72
1 month, median (IQR)	4 (3–6)	4 (3–6)	0.68
3 months, median (IQR)	2 (1–3)	2 (1–3)	0.81
Within-group P-value^‡^	< 0.001	< 0.001	—

^†^Mann–Whitney U test

^‡^Friedman test

### Secondary outcome: headache severity

At baseline, headache severity distributions were similar between the two groups (*P* = 0.77, Mann–Whitney U test). Most participants reported moderate to severe headaches at study entry. Following treatment initiation, both groups experienced significant improvements in headache severity. In the cinnarizine group, the median headache severity score decreased from 2 (IQR:2–3) at baseline to 2 (IQR:1–2) at one month and to 1 (IRQ: 1–2) at three months (*P* < 0.001, Friedman test). In the topiramate group, the median severity scores similarly declined from 2 (IQR:2–3) to 2 (IQR:1–2) and 1 (IQR:1–2) at the respective follow-up points (*P* < 0.001). No statistically significant differences in headache severity scores were observed between the two groups at one month (*P* = 0.59) or three months (*P* = 0.74). The between-group effect size at three months was negligible (*r* = 0.04) (Table 3).

**Table 3:** Headache severity scores over the study period

Time point	Cinnarizine (*n* = 48) Median (IQR)	Topiramate (*n* = 48) Median (IQR)	*P*-value^†^
Baseline	2 (2–3)	2 (2–3)	0.77
1 month	2 (1–2)	2 (1–2)	0.59
3 months	1 (1–2)	1 (1–2)	0.74
Within-group P-value^‡^	< 0.001	< 0.001	—

Severity score: 1 = mild, 2 = moderate, 3 = severe

^†^Mann–Whitney U test

^‡^Friedman test

### Categorical changes in headache frequency and severity

By the end of the 12-week treatment period, the proportion of participants experiencing fewer than four migraine attacks per month increased substantially in both groups. In the cinnarizine group, 79.2% of participants reported fewer than four attacks per month compared with 77.1% in the topiramate group (*P* = 0.968, Fisher’s exact test).

Similarly, at three months, mild headaches were reported by 66.7% of participants in the cinnarizine group and 64.6% in the topiramate group, while no participants in either group reported severe headaches. The distribution of headache severity categories did not differ significantly between the groups (*P* = 0.88) (Table 4).

**Table 4:** Distribution of headache frequency categories at three months

Frequency category	Cinnarizine (*n* = 48)	Topiramate (*n* = 48)	*P*-value*
< 2 attacks/month	18 (37.5%)	17 (35.4%)	
2–4 attacks/month	20 (41.7%)	20 (41.7%)	
5–8 attacks/month	10 (20.8%)	11 (22.9%)	
> 8 attacks/month	0 (0%)	0 (0%)	0.968

*Fisher’s exact test

### Adverse events and tolerability

Both medications were generally well tolerated, and no serious adverse events requiring treatment discontinuation were reported. Mild-to-moderate adverse events occurred in 27.1% of participants in the cinnarizine group and 35.4% of participants in the topiramate group, although this difference was not statistically significant (*P* = 0.509).

The most frequently reported adverse events included fatigue or sedation (14.6% in the cinnarizine group vs. 18.8% in the topiramate group; *P* = 0.793) and daytime drowsiness (12.5% vs. 10.4%, respectively; *P* = 1.0). Appetite reduction was reported less frequent in the cinnarizine group compared with the topiramate group (4.2% vs. 14.6%), though this difference was not statistically significant (*P* = 0.159, Fisher’s exact test; OR = 0.255, 95% CI: 0.05–1.27). Adverse events were reported and compared separately for each treatment group (Table 5).

**Table 5:** Reported adverse events during the study period

Adverse event	Cinnarizine (*n* = 48)	Topiramate (*n* = 48)	*P*-value*
Any adverse event	13 (27.1%)	17 (35.4%)	0.509
Fatigue / sedation	7 (14.6%)	9 (18.8%)	0.793
Daytime drowsiness	6 (12.5%)	5 (10.4%)	1.0
Appetite reduction	2 (4.2%)	7 (14.6%)	0.159
Serious adverse events	0 (0%)	0 (0%)	—

*Fisher’s exact test

## Discussion

In this randomized clinical trial, both cinnarizine and topiramate demonstrated significant efficacy in reducing the frequency and severity of migraine attacks in children over a 12-week treatment period. The results showed a substantial and clinically meaningful improvement in migraine outcomes within each treatment group, with no statistically significant differences detected between the two medications in terms of primary or most secondary efficacy endpoints. This study was not powered for equivalence or non-inferiority testing; therefore, we cannot conclude that the treatments are comparable or equivalent. The observed small between-group difference may reflect a Type II error due to limited power for detecting small effect sizes. Furthermore, the high prevalence of family history of migraine in our cohort (83.3%), which is consistent with established international literature, reinforces the clinical validity of the diagnosis and the genetic basis of the disorder within the studied sample.

The observed reduction in monthly migraine attack frequency in both groups is consistent with previous studies evaluating preventive therapies for pediatric migraine. Topiramate has been widely studied and is considered one of the most effective prophylactic agents for migraine in children and adolescents, with multiple randomized controlled trials demonstrating its ability to significantly reduce migraine frequency and headache-related disability [4, 10, 15]. The present findings further support the role of topiramate as an effective preventive agent, while also highlighting that comparable outcome can be achieved with alternative medications such as cinnarizine.

Cinnarizine, an antihistamine with calcium channel–blocking properties, has been investigated less extensively in pediatric migraine populations; however, existing evidence suggests favorable efficacy and tolerability. Previous randomized trials have reported that cinnarizine is effective in reducing migraine frequency and severity in children and adolescents, with efficacy comparable to propranolol or topiramate [16, 17]. Similarly, a recent randomized double-blind trial comparing cinnarizine with amitriptyline demonstrated significant improvements in migraine outcomes with cinnarizine, supporting its role as a viable preventive option in pediatric migraine management [14]. The present study aligns with these findings and adds further evidence by directly comparing cinnarizine with topiramate in a pediatric population.

Regarding headache severity, both treatment groups experienced significant improvements over time, with the majority of participants reporting mild headaches by the end of the study and no cases of severe headache persisting at three months. This pattern of improvement is clinically relevant, as headache severity is a major determinant of functional impairment and quality of life in children and adolescents with migraine. The absence of a significant difference between the two groups suggests that both medications are similarly effective in mitigating the overall burden of migraine-related pain.

An important aspect of pediatric migraine prophylaxis is medication tolerability, given the potential impact of adverse events on treatment adherence and long-term outcomes. In the present study, both cinnarizine and topiramate were generally well tolerated, with no serious adverse events reported and no statistically significant differences in overall adverse event rates. Appetite reduction was numerically more frequent in the topiramate group, consistent with its known side-effect profile, but the difference did not reach statistical significance in this sample. This finding is consistent with the well-documented side effect profile of topiramate, including weight loss and appetite suppression [15, 18]. In contrast, cinnarizine showed a numerical trend toward a lower incidence of appetite reduction compared with topiramate (4.2% vs. 14.6%). Although this difference was not statistically significant in our sample, this trend aligns with the drug’s expected metabolic profile and may be particularly advantageous in children and adolescents, where growth and nutritional status are critical considerations.

The favorable tolerability profile of cinnarizine observed in this study supports previous reports suggesting that it may have fewer cognitive and metabolic side effects compared with antiepileptic drugs used for migraine prophylaxis [16, 19]. Given concerns regarding cognitive impairment, academic performance, and weight changes in children receiving long-term preventive therapy, cinnarizine may represent a suitable alternative for patients who are unable to tolerate topiramate or in whom such side effects are of particular concern. Factors such as individual patient characteristics (e.g., weight and age) should be considered when prescribing, as fixed dosing regimen used here may not represent optimal individualized therapy and could have influenced the observed between-group comparisons.

### Limitations

Despite the strengths of this study, including its randomized design and prospective data collection, several limitations should be acknowledged. First, we utilized a fixed dosing regimen (25 mg/day for both drugs) rather than weight-adjusted dosing (mg/kg). This represents a significant methodological limitation in a pediatric population, as optimal dosing for topiramate is typically 1–2 mg/kg/day (and sometimes higher with titration), while cinnarizine dosing in prior pediatric trials has also been weight-based (e.g., 1.5 mg/kg/day). The fixed dose may have been suboptimal or sub-therapeutic for older/heavier children and adolescents, potentially confounding between-group comparisons of efficacy and preventing interpretation of the study as a true comparison at optimal dosing for each drug. Although both groups showed significant within-group improvement, any between-group conclusions must be interpreted with caution due to this issue. Additionally, any changes of body weight and height of participants during the 12-week therapy were not systematically assessed. Given the potential appetite-stimulating and weight-gain effects of cinnarizine and the appetite-suppressing and weight-loss effects of topiramate, the absence of these anthropometric data limits the full evaluation of the metabolic and nutritional impact of the two treatments. We used migraine attack frequency rather than monthly migraine days as the primary endpoint. While attack frequency is a clinically relevant measure, it limits direct comparability with the majority of recent trials that report migraine days, thereby reducing the external validity and ease of comparison with current literature. The study duration was limited to 12 weeks, which restricts the ability to draw conclusions regarding the long-term efficacy and safety of both medications. Additionally, although headache frequency and severity were systematically assessed, the study did not incorporate standardized quality-of-life assessment tools or validated severity scales like PedMIDAS, which could provide a more comprehensive evaluation of treatment impact. Furthermore, the absence of a placebo control group represents a major limitation. Given the well-documented high placebo response rate in pediatric migraine trials (often exceeding 30%), the observed improvements in both groups cannot be definitively attributed to the pharmacological effects of the interventions. This significantly limits causal inference and the ability to distinguish true drug effects from placebo response. The study was powered for moderate effect sizes (Cohen’s d = 0.6). The fact that no statistically significant differences were observed for the primary outcome (*P* = 0.81) suggests that the study may have been underpowered to detect the observed small between-group differences, thus increasing the risk of a Type II error.

Future research should focus on larger, multicenter randomized controlled trials with longer follow-up periods to further evaluate the comparative effectiveness and long-term safety of cinnarizine and topiramate in pediatric populations. Incorporating patient-reported outcomes, cognitive assessments, and quality-of-life measures would also enhance understanding of the broader clinical impact of these treatments. Such studies could contribute to the development of more individualized, evidence-based strategies for pediatric migraine prophylaxis.

## Conclusion

In conclusion, both cinnarizine and topiramate were associated with significant reductions in the frequency and severity of migraine attacks over 12 weeks. No statistically significant differences were observed between the two medications in efficacy outcomes.

Although a numerical trend toward lower appetite-related adverse events was observed with cinnarizine, this difference was not statistically significant. Given the sample size and the fact that the study was not designed as an equivalence or non-inferiority trial, larger adequately powered studies are required before any conclusions regarding relative efficacy or tolerability advantages can be drawn.

## Supplementary Information

Supplementary Material 1.

Supplementary Material 2.

## Data Availability

The dataset generated and analyzed during the current study are available from corresponding author on reasonable request.
