# Supplementary material for: Cinnarizine vs. topiramate for migraine prophylaxis in children: a randomized, double-blind, parallel-group clinical trial
Source: BMC Neurol. 2026 May 9;26:348. doi: 10.1186/s12883-026-04950-1 (PMC13196016; doi:10.1186/s12883-026-04950-1)
Supplement: Supplementary file 1 — Supplementary Material 1. [file 12883_2026_4950_MOESM1_ESM.pdf]

**Section / Item**	**Item No.**	**Description**	**Location in Manuscript (Section/Page)**
**Title & Abstract**	**1a**	Identification as a randomised trial.	Title
	**1b**	Structured summary of trial design, methods, results, and conclusions.	Abstract
**Open Science**	**2**	Trial registry name and identifying number (with URL).	Ethical Considerations (IRCT)
	**3**	Where the protocol and statistical analysis plan can be accessed.	Availability of Data and Materials
	**4**	Where and how participant data and statistical code can be accessed.	Availability of Data and Materials
**Funding & COI**	**5a**	Sources of funding and role of funders.	Funding Section
	**5b**	Financial or non-financial conflicts of interest of authors.	Conflicts of Interest Section
**Introduction**	**6**	Scientific background and explanation of rationale.	Introduction
	**7**	Specific objectives regarding both benefits and harms.	End of Introduction
**Methods**	**8**	Patient or public involvement in the design and conduct.	Not reported.

Section / Item	Item No.	Description	Location in Manuscript (Section/Page)
	**9**	Trial design (e.g., parallel, allocation ratio) and framework.	Study Design (Parallel, 1:1 ratio)
	**10**	Important changes to methods after trial commencement.	None reported.
	**11**	Settings and locations where the data were collected.	Study Design (Urmia, Iran)
**Eligibility**	**12a**	Eligibility criteria for participants.	Participants Section
	**12b**	Eligibility criteria for centers and those performing interventions.	Study Design (Specialized clinic)
**Interventions**	**13**	Details of interventions and comparator to allow replication.	Interventions Section
**Outcomes**	**14**	Pre-specified primary and secondary outcomes and timing.	Outcome Measures
	**15**	How harms were defined and assessed.	Outcome Measures / Adverse Events
**Sample Size**	**16a**	How sample size was determined and assumptions.	Sample Size Calculation
	**16b**	Interim analyses and stopping guidelines.	Not applicable.

Section / Item	Item No.	Description	Location in Manuscript (Section/Page)
**Randomization**	**17a**	Method used to generate the random allocation sequence.	Randomization (Variable block size)
	**17b**	Type of randomization and details of any restriction.	Randomization (Block randomization)
	**18**	Mechanism used to implement the allocation (concealment).	Randomization (Opaque sealed envelopes)
	**19**	Who generated the sequence and enrolled/assigned participants.	Randomization (Independent researcher)
**Blinding**	**20a**	Who was blinded after assignment to interventions.	Randomization (Double-blind)
	**20b**	Description of the similarity of interventions for blinding.	Randomization (Identical appearance)
**Statistical Methods**	**21a**	Statistical methods used to compare groups for outcomes.	Statistical Analysis
	**21b**	Definition of the population analyzed in each group.	Participant Flow / Results
	**21c**	How missing data were addressed.	Results (No loss to follow-up)

Section / Item	Item No.	Description	Location in Manuscript (Section/Page)
	**21d**	Methods for additional analyses (subgroup/sensitivity).	Statistical Analysis
**Results**	**22a**	Number of participants assigned and analyzed.	Participant Flow / Results
	**22b**	Losses and exclusions after randomization and reasons.	Participant Flow (None)
	**23a**	Dates defining the periods of recruitment and follow-up.	Study Design (Year 2023)
	**23b**	Why the trial ended or was stopped early.	Not applicable.
**Intervention Delivery**	**24a**	Fidelity: how the intervention was actually delivered.	Follow-Up Section
	**24b**	Concomitant care and interventions that were permitted.	Interventions (Rescue medication)
**Baseline Data**	**25**	A table showing baseline demographic and clinical features.	Table 1
**Outcomes & Harms**	**26**	For each outcome, results for each group and effect size.	Results / Tables 2, 3, 4
	**27**	All important harms or unintended effects in each group.	Adverse Events / Table 5

Section / Item	Item No.	Description	Location in Manuscript (Section/Page)
	**28**	Any other analyses performed (subgroup/post hoc).	None reported.
**Discussion**	**29**	Interpretation consistent with results (balance of benefits/harms).	Discussion
	**30**	Trial limitations (potential bias, imprecision, generalizability).	Discussion (Limitations)
