# Supplementary material for: Cinnarizine vs. topiramate for migraine prophylaxis in children: a randomized, double-blind, parallel-group clinical trial
Source: BMC Neurol. 2026 May 9;26:348. doi: 10.1186/s12883-026-04950-1 (PMC13196016; doi:10.1186/s12883-026-04950-1)
Supplement: Supplementary file 2 — Supplementary Material 2. [file 12883_2026_4950_MOESM2_ESM.pdf]

Table 1. Personal Information

Variable	Value
1. Full Name	
2. Date of Birth	
3. Place of Birth	
4. Mobile Number	
5. Current Residence	
6. Education Level	
7. National ID	

Table 2. Past Medical History (PMH)

Item	Description
**Surgical History**	
**Neurological Examination**	☐ Normal / ☐ Abnormal: ___________
**Other Neurological Disorders**	☐ Yes (Specify: Epilepsy, Stroke, etc.) / ☐ No

Table 3. Family History of Migraine

Relative	History of Migraine	Relative	History of Migraine
**Father**	☐ Yes / ☐ No	**Mother**	☐ Yes / ☐ No
**Sister**	☐ Yes / ☐ No	**Brother**	☐ Yes / ☐ No
**Uncle (Paternal)**	☐ Yes / ☐ No	**Aunt (Paternal)**	☐ Yes / ☐ No
**Uncle (Maternal)**	☐ Yes / ☐ No	**Aunt (Maternal)**	☐ Yes / ☐ No
**Grandfather**	☐ Yes / ☐ No	**Grandmother**	☐ Yes / ☐ No

Table 4. Headache Diagnosis

Category	Options
**Initial Diagnosis**	☐ Migraine / ☐ Tension / ☐ Cluster / ☐ Chronic / ☐ Migraine Variants
**Final Diagnosis**	☐ Migraine with Aura / ☐ Migraine without Aura / ☐ Tension / ☐ Cluster / ☐ Chronic / ☐ Migraine Variants

Table 5. Detailed Headache Characteristics (Baseline)

Feature	Options
**1. Time of Onset**	☐ < 6 months / ☐ 6–12 months / ☐ > 12 months
**2. Pain Location**	**Unilateral:** ☐ Frontal ☐ Parietal ☐ Occipital ☐ Temporal ☐ Generalized<br><br>**Bilateral:** ☐ Frontal ☐ Parietal ☐ Occipital ☐ Temporal ☐ Generalized
**3. Severity**	☐ Mild / ☐ Moderate / ☐ Severe / ☐ Disabling
**4. Nature of Pain**	☐ Pressing / ☐ Stabbing / ☐ Burning / ☐ Pulsating / ☐ Vague
**5. Duration of Attack**	☐ Minutes / ☐ Hours / ☐ Days
**6. Attack Frequency**	☐ < 1 per month / ☐ 2–4 per month / ☐ 5–8 per month / ☐ > 9 per month
**7. Diurnal Pattern**	☐ Morning / ☐ Noon / ☐ Afternoon / ☐ Evening / ☐ Night / ☐ No difference
**8. Seasonal Pattern**	☐ Spring / ☐ Summer / ☐ Autumn / ☐ Winter / ☐ No difference
**9. Aura Symptoms**	☐ None / ☐ Visual / ☐ Auditory / ☐ Sensory / ☐ Brainstem symptoms
**10. Warning Signs**	☐ Mood changes / ☐ Personality changes / ☐ Appetite changes / ☐ Fatigue / ☐ Neck pain
**11. Associated Symptoms**	☐ Nausea / ☐ Vomiting / ☐ Dizziness / ☐ Tearfulness / ☐ Restlessness / ☐ Photophobia / ☐ Phonophobia
**12. Relieving Factors**	☐ Sleep / ☐ Rest in dark room / ☐ Exercise / ☐ Scalp massage / ☐ Bandaging / ☐ Cold/Warm compress
**13. Impact on Function**	☐ Normal / ☐ Mild effect / ☐ Severe effect / ☐ Disabling

Table 6. Diagnostic Results & Medications

Evaluation	Normal	Abnormal	Not Performed
**Routine Lab Tests**	☐	☐	☐
**EEG**	☐	☐	☐
**Brain CT Scan**	☐	☐	☐
**Brain MRI**	☐	☐	☐

Table 7. Medication History

Medication History	Details
Current Abortive Meds	
Prior Prophylactic Meds	
